# Community perceptions and socio-economic implications of conservation corridors and networks in the Vhembe District, Limpopo, South Africa

**DOI:** 10.1007/s13280-024-02073-w

**Published:** 2024-09-21

**Authors:** Alexandra Dalziel, Mary Evans

**Affiliations:** University of the Witwatersrand, 1 Jan Smuts Avenue, Braamfontein, Johannesburg, South Africa

**Keywords:** Community engagement, Conservation corridors, Ecological networks, Protected areas

## Abstract

Social facets linked to conservation corridors and ecological networks have received relatively limited academic attention. This study explores the perspectives of researchers, NGO representatives, and landowners, as well as the community’s ideas of conservation efforts and corridor potential in the Vhembe District, Limpopo, South Africa. Surveys and interviews were conducted with communities, regional stakeholders, and landowners. The findings revealed that the community participants strongly support corridor implementation. The results indicate that this support is driven by the anticipated socio-economic benefits in the form of jobs. However, the employment opportunities might not align with the resident's expectations. The study identifies several challenges to corridor establishment, including infrastructure and financial constraints. Moreover, the findings revealed a lack of supportive legislation and highlighted concerns over protected area's accessibility. The study contributes to the global academic discourse by emphasizing the importance of community engagement before corridor and network implementation. It also addresses the complex trade-offs inherent in such projects, regardless of location. The methodological approach employed in this research transcends its regional context and offers actionable insights applicable worldwide.

## Introduction

Conservation corridors and ecological networks, grouped under the term landscape initiatives in this paper, are among the most popular strategies for biodiversity conservation as habitat fragmentation and biodiversity loss continue to increase. Conservation corridors are contiguous areas of relatively natural land that link protected areas, facilitating species mobility, dispersal, and in-situ protection (Rouget et al. 2006; Beier 2018). Similarly, ecological networks aim to mitigate the negative effects of fragmentation by encompassing large areas of vegetation, including numerous protected areas and conservation corridors (Pryke and Samways 2012). Ecological research consistently underscores the beneficial impact of increased habitat connectivity facilitated by corridors on species conservation and ecological functions (Hanski and Ovaskainen 2000; Bennett and Mulongay, 2006; Crooks and Sanjayan, 2006; Haddad et al*.* 2014; Hilty et al*.* 2019; Joubert van der Merwe et al*.* 2019; Hilty et al*.* 2020; Battisti 2023). Realizing a corridor's potential to connect and benefit surrounding communities is contingent upon thorough community involvement (Goldman 2009; Kikoti et al. 2010; Townsend and Masters 2015). While public engagement is difficult to guarantee, public hearings, community projects, development planning, NGO engagement (Bennett 2004), and consensus within communities are imperative to ensure the corridors or networks' feasibility and guarantee the fulfillment of environmental and local needs (Rosenthal et al*.* 2012).

Global examples underscore the importance of community engagement in conservation initiatives such as corridors and networks (Adhikari et al*.* 2007; Townsend and Masters, 2007; Kikoti et al*.* 2010; Hemp and Hemp 2018; Mbane et al*.* 2019). For instance, in the Terai Arc landscape of Nepal, the utilization of community forests allowed the community to use the land, control some of the forested areas, and foster a sense of ownership. At the same time, environmental goals were achieved (Adhikari et al*.* 2007). In some cases, community engagement mitigated conflict and enhanced forest management and environmental conditions (Birch et al. 2014). However, the sustained success of such initiatives hinges upon ongoing community involvement, as exemplified by the cautionary example of the Kitenden corridor in the Kilimanjaro region of Kenya (see Kikoti et al*.* 2010; Hemp and Hemp 2018). Despite initial local support, the corridor's integrity was jeopardized over time due to land use tenure changes and encroachment, leading to heightened human-wildlife conflicts (Hemp and Hemp 2018; Mbane et al*.* 2019).

Community engagement emerges as a linchpin in the efficacy of conservation corridors and networks, fostering a sense of collective responsibility and stewardship among locals (Townsend and Masters, 2007; Kikoti et al*.* 2010). By actively involving communities, a harmonious coexistence between local land use activities and environmental conservation goals can be achieved (Lombard et al*.* 2010).

Dalziel and Evans (2024) noted a deficiency in the protected area estate of the Vhembe District in Limpopo Province, South Africa. Over 10,000 km^2^ of essential Key Biodiversity Areas remain unprotected. The authors recommend establishing corridors and networks in the region to increase the coverage of essential ecosystems. However, comprehensive assessments of community's land use, expected benefits, and conservation perceptions are imperative before introducing such landscape initiatives.

Taking the Vhembe District in Limpopo, South Africa, as an illustrative case study, we conducted a preliminary screening assessment. We used questionnaires and interviews to assess attitudes toward landscape initiatives among residents and to uncover if and what benefits were expected from such a project. This approach identified landscape initiatives' potential social benefits, drawbacks, and applicability. These findings exceed the regional context, as community engagement is framed as a critical factor when establishing corridors.

## Materials and methods

### Study area

**Fig. 1:**
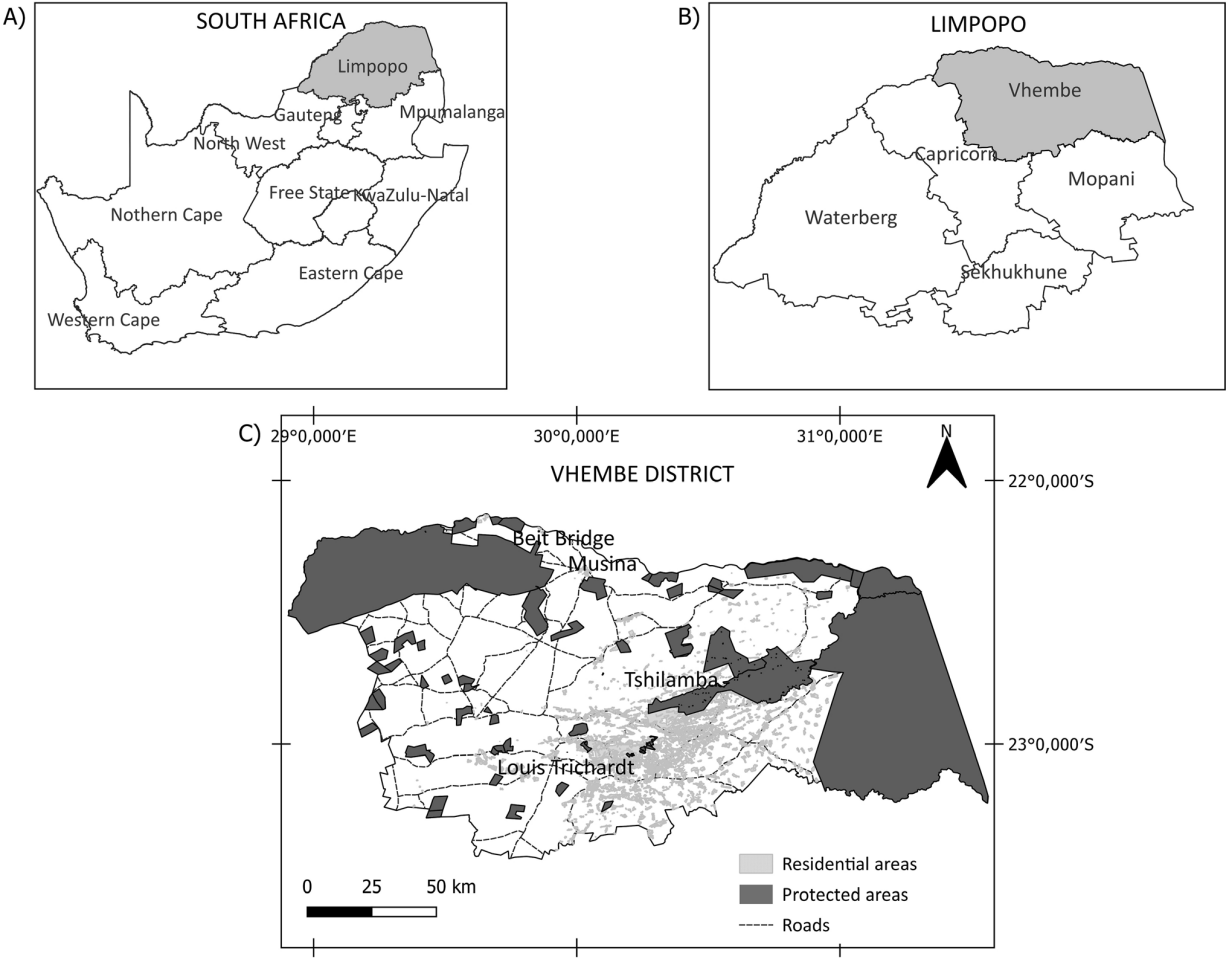
Map of the Vhembe District in the northern part of Limpopo, South Africa. It illustrates the location of the major urban centers, road networks, and the dispersion of the protected areas

The study was conducted in the Vhembe District located in the Limpopo province in South Africa (22° 46′ 10″ S, 29° 58′ 26″ E Fig. 1). Encompassing an area of 25, 596 km^2^, the Vhembe District is the most densely populated among the districts in Limpopo, housing approximately 24.3% of the provincial population (Limpopo Provincial Government 2023). The latest estimates put the population of the Vhembe District at around 1.4 million people (Limpopo Provincial Government 2023). Predominant economic activities include mining, retail, finance, and community services. The district has a high unemployment rate of 43%. A sizable portion of its inhabitants live in poverty, as 51% of the population falls in the Upper-Bound Poverty Line (UBPL) (Limpopo Provincial Government 2023). The UBPL refers to the food poverty line, and individuals living on around ZAR 1 335 per month, or just over $2 a day, fall within this poverty line and can purchase basic food and non-food items.

The Vhembe District contains 68 protected areas, of which 46 are privately owned. The average size of the private protected areas in the Vhembe District is around 25 km^2^ (Dalziel and Evans 2024). Additionally, the Vhembe District is home to two major protected areas, the Kruger National Park and Mapungubwe National Park, a UNESCO-declared World Heritage Site (Fig. 1). Encompassing an impressive 38% of the district’s landmass, these protected areas collectively contribute to 7% of South Africa’s national protected area estate (Dalziel and Evans 2024). The Soutpansberg Mountain range runs through the district and is recognized as a center of endemism in South Africa. This region is the habitat for 22 endemic taxa (Hahn 2019). It has three proclaimed Key Biodiversity Areas (KBA), the Kruger National Park KBA, the Mapungubwe National Park KBA, and the Soutpansberg KBA. KBAs are defined as “sites of significance for the global persistence of biodiversity” (IUCN 2016, pg. 8). Despite their biological significance, large sections of the KBAs are unprotected in the region (Dalziel and Evans 2024). This low degree of protection is a global phenomenon, as only half of the world’s KBA sites are protected (Kullberg et al. 2019).

### Research method

To understand the perceptions of the local community and stakeholders regarding landscape initiatives and conservation efforts in the region, we employed methodological triangulation, which uses multiple methods to maximize the validity of fieldwork (Denzin 1970). Our study adopted a mixed-method approach involving interviews with ecological researchers and critical stakeholders. Additionally, representatives from conservation organizations such as SANParks, PeaceParks, African Parks, Boundless South Africa, and landowners were interviewed. Furthermore, to capture the sentiments and viewpoints of the community, we administered and collected surveys.

The University's of the Witwatersrand’s Ethics Committee (non-medical) granted ethics clearance, and the ethics clearance number for the study is H21/04/07. The study was ruled low-risk as we did not approach anyone in the vulnerable person category or anyone under 18 years old.

#### Interviews

We conducted interviews with stakeholders, totaling 15 participants, including academics and researchers (*n* = 5), NGO representatives (*n* = 7), and landowners (*n* = 3). In a previous study, we identified academics and researchers through a comprehensive literature review on corridors and networks in South Africa (Dalziel and Evans 2023). The principal contributors to the body of literature on corridors and ecological networks were subsequently contacted, along with the primary NGOs involved in corridor initiatives in South Africa. Furthermore, landowner details were obtained through pre-existing contacts, although only three landowners responded to the outreach efforts. We used open-ended, semi-structured interviews raising key thematic points pertaining to conservation practices and essential considerations preceding implementing landscape initiatives. Questions specifically focused on the potential and applicability of landscape initiatives in South Africa and the Vhembe District. Interviews were conducted over the phone or Microsoft Teams sessions between March and August 2022. We then transcribed the interviews using a thematic analysis approach (Vaismoradi et al. 2013).

#### Surveys

We also distributed surveys to community members in the Vhembe District. The district’s population is 1 402 779 individuals, with 748 128 falling within the age bracket of 18 to 60. A representative sample of 384 participants was calculated from this population, ensuring a 5% margin of error and a 95% confidence level. Data were collected between June and August 2023. Respondents were approached using simple random sampling, where individuals are chosen to ensure each person has an equal chance of selection (Noor et al. 2022). Local assistants, residents of the surveyed towns, assisted in translating and explaining the survey questions as they were distributed throughout the towns and rural villages, including Louis Trichardt, Khubvi, and Thohoyandou. Depending on their understanding of the questions, participants filled in the surveys themselves or were assisted by the research team.

Data collection stopped after reaching the predetermined sample size. The survey encompassed inquiries about land use, comprehension of conservation concepts, and questions about how often the person visited the area’s nature reserves. The questionnaire also included questions regarding the potential benefits of linking such reserves and the anticipated advantages thereof.

## Results

### Benefits of landscape initiatives and their potential in the Vhembe District

Among the 15 interview participants, including landowners, researchers, and NGO representatives, 73% felt that traditional protected areas were no longer effective in mitigating habitat fragmentation and biodiversity loss at present rates. Their dissatisfaction stems from various shortcomings of enclosed conservation areas. Landowners felt that protected areas had limited coverage and decreased species migration (Table 1). Researchers and NGO representatives added to this and felt that protected areas isolated populations and contributed to landscape fragmentation (Table 1). Moreover, 12 of the 15 participants (80%) advocated for an alternative approach of connecting protected areas through corridors as a more viable solution to mitigate habitat fragmentation and biodiversity loss. The respondents notably endorsed this strategy’s potential applicability in South Africa. The respondents attributed this sentiment to the plethora of benefits associated with corridors and networks, including increasing migration and protected area coverage, providing resilience to existing protected areas, and effectively protecting vulnerable species and ecosystems (Table 1).

**Table 1:** Participant perspectives on reasons for protected area ineffectiveness and landscape initiative effectiveness. Participants were allowed to select multiple answers

Participant type	Number of responses (*n* = 11)	Reasons for protected area ineffectiveness	Number of responses (*n* = 12)	Reasons for landscape initiative effectiveness
Landowners	2	Inadequate coverage (2), limited species migration (1)	2	Increase in protected area coverage (1), increased migration (2)
Researchers	4	Inadequate coverage (3), limited species migration (3), isolating genetic populations (4)	4	Increase in protected area coverage (3), increased migration (4), genetic mixing (4), provide resilience to ecosystems and species (1)
NGO representatives	5	Inadequate coverage (2), limited species migration (5), isolating genetic populations (3), furthering the fragmentation of landscapes (1)	6	Increase in protected area coverage (5), increased migration (4), genetic mixing (3)

Most participants (80%) were ‘very familiar’ with the Vhembe District, and 83% believed a corridor or network would benefit the area. This sentiment was motivated by the area’s geography, characterized by large strips of undeveloped land deemed ideal for a corridor. Furthermore, three interviewees noted the potential socio-economic advantages, such as job creation, that could benefit the local community through conservation corridors or networks.

### Factors hindering corridor and network implementation

Interviews suggested a preference for landscape initiatives and their potential benefits for South Africa and the Vhembe District. Specific factors must be considered before implementing corridors and networks. Foremost is land ownership. When asked if private and national protected areas should be linked, 9 of the 15 interviewees (60%) said no and cited jurisdiction issues as a hindrance, as land rights would be questioned.

**Fig. 2:**
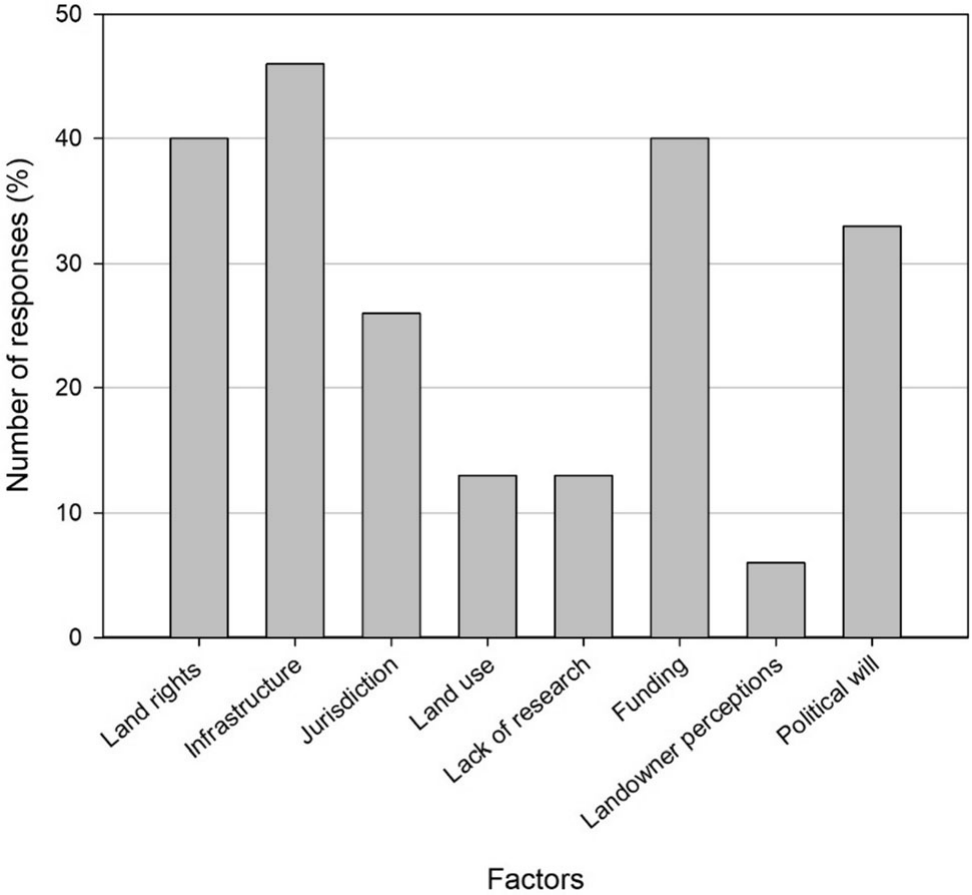
Factors hindering protected area expansion and corridor and network implementation. Findings were derived from interviews (*n* = 15)

Results underscore additional potential obstacles associated with landscape initiatives and the expansion of protected areas (Fig. 2). Infrastructure, in the form of roads or ongoing developments and human settlements, land rights, funding, and political will emerged as a recurring theme throughout the interviews (Fig. 2).

Moreover, corridors and networks must meet specific physical parameters to conserve and capture vulnerable ecosystems effectively. The first parameter involves the optimal width of the corridor, a topic often debated in conservation corridor literature (Andreassen et al. 1996; Beier 2018; Ford et al. 2020; van Schalkwyk et al. 2020). Concerning Table 2, three of the 15 participants (20%), all NGO representatives, felt corridors should be between 300 and 500 m. Additionally, 20% of the participants, one researcher, a landowner, and an NGO representative advocated for corridors between 1 and 2 km. Most participants (33%) advocated for corridors over 2 km. This sentiment was not shared by any landowners (Table 2).

**Table 2:** Interview responses regarding the optimal corridor width and the preferred number of protected areas in ecological networks (*n* = 15)

Parameters	Interval	Stakeholder type	Percentage (%)
Width	50 m–100 m	Researcher	6.6
	100 m–300 m	Landowner	6.6
	300 m–500 m	NGO	20
	500 m to 1 km	Researcher, landowner	13.3
	1 km to 2 km	Landowner, researcher, NGO	20
	Over 2 km	NGO, researcher	33.3
Number of protected areas	1–2	Landowner, researcher	20
	2–3	Researcher	13.3
	3–4	NGO	20
	More than 4	Landowner, NGO, researcher	46.6

The second important parameter concerns the optimal number of protected areas included within a network. Seven respondents (46%) stated that for corridors to be effective, they should ideally incorporate more than four protected areas. The necessity of connecting multiple conservation areas was unanimously shared across all stakeholders (Table 2).

Based on these few interviews, there seems to be a preference for wider corridors and networks with multiple protected areas. However, as one participant stated:

*“It is very difficult to say exactly what the optimal corridor and network layout should be as landscapes vary. Of course, you want wide corridors and your networks to include as many protected areas as possible, but this is not always possible. What works in one area may not work in another”.* (Pers. Comm, 2023a, researcher).

### Community demographics and land use

**Fig. 3:**
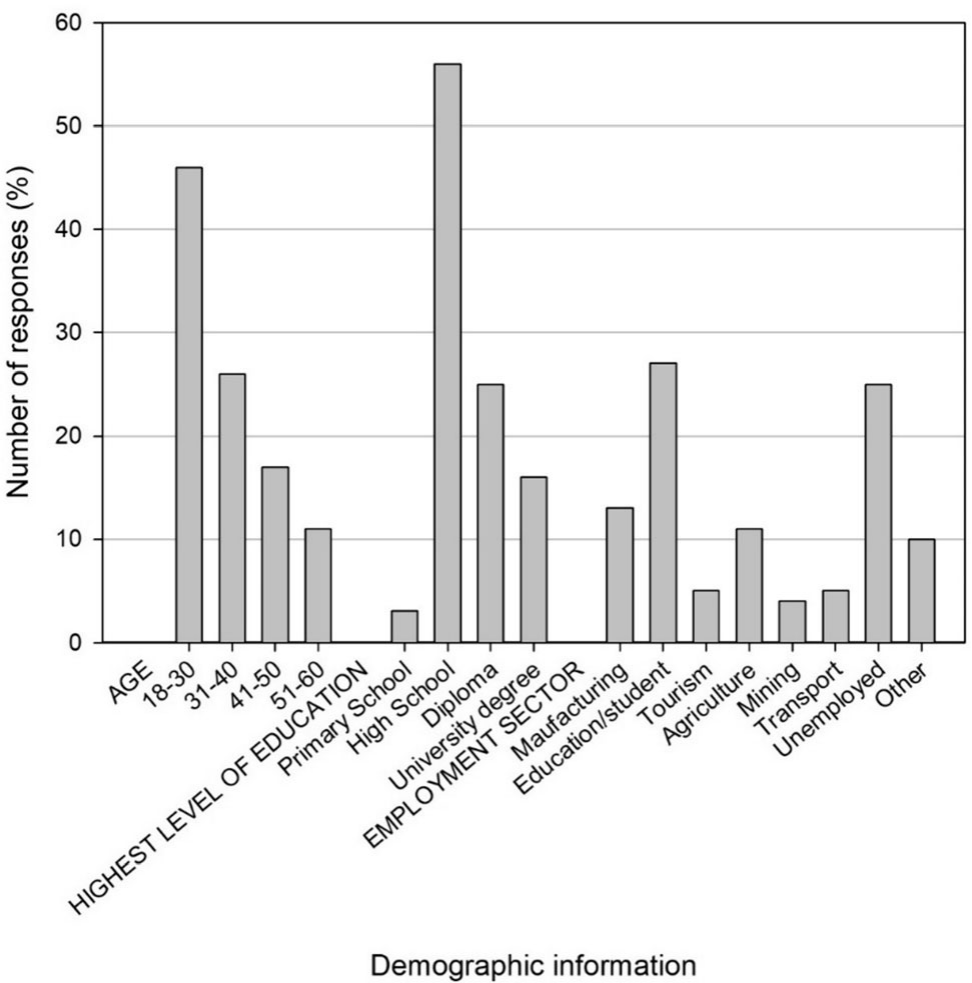
Demographic information including age, highest education level, and employment sector obtained from surveys (*n* = 384)

In addition to conducting stakeholder interviews, 384 surveys were distributed to local community members living in the Vhembe District to gauge their perceptions of conservation and corridors. Most participants, 46%, were between 18 and 30 years old, with the second highest percentage (26%) falling between the 31- and 40 year-old age bracket (Fig. 3). The majority of participants (56%) had finished high school as their highest level of education, with 25% holding a diploma. The occupation distribution revealed that 27% of participants were students, and 25% were unemployed (Fig. 3).

**Fig. 4:**
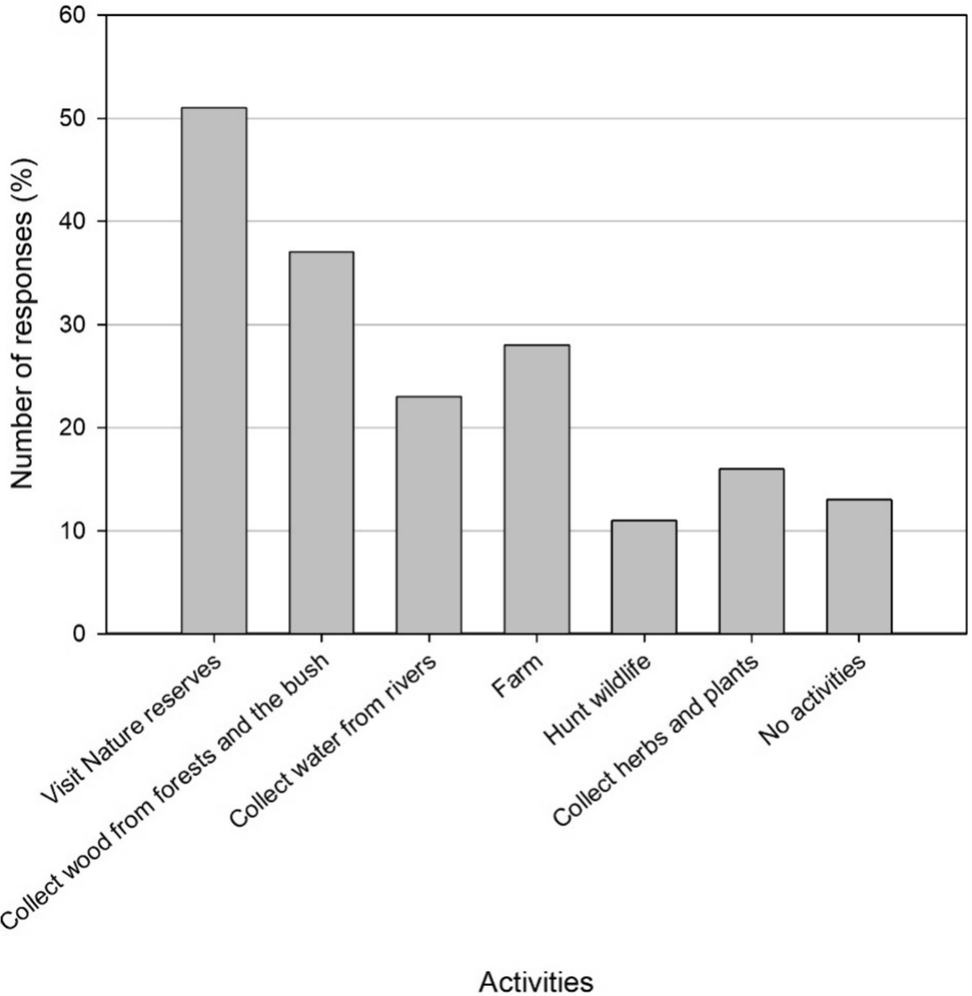
Activities conducted in the Vhembe District according to 384 participants

The respondents were residents of the town itself or the suburbs and rural areas around it, dwelling itself is a form of resource and land use. However, we asked questions about land use activities to ascertain the range of activities conducted in the area. Most participants (87%) selected one or more activities listed (Fig. 4). The top four responses included (1) visiting nature reserves (51%), (2) collecting wood from the bush/veld (37%), (3) farming (28%), and (4) collecting water from rivers (23%) (Fig. 4). Merely 13% of participants stated they did not engage in any of the listed activities (Fig. 4). However, we contend that living in an area constitutes a form of land use, as it involves occupying space.

### Community perceptions of conservation and protected areas

**Fig. 5:**
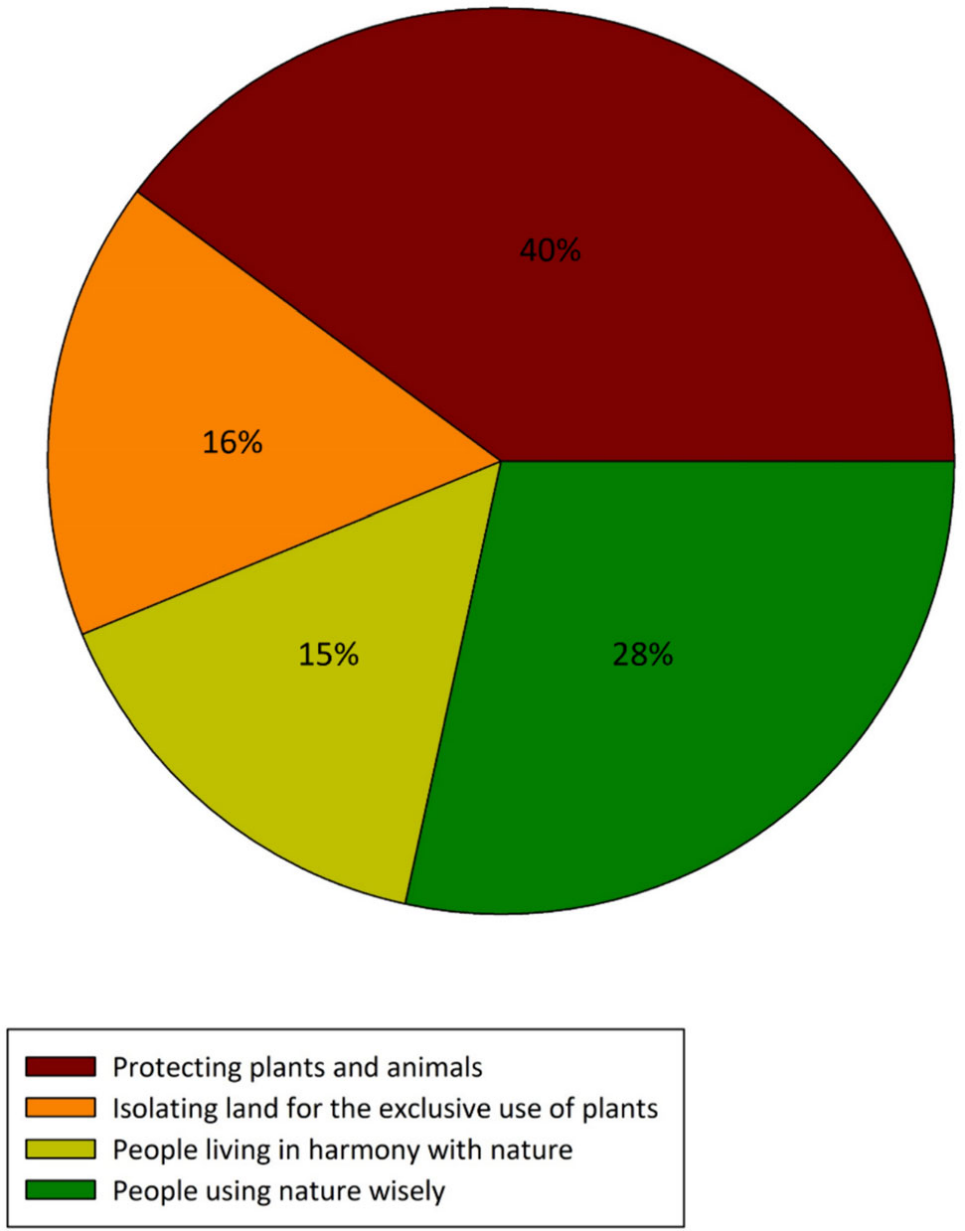
Community member’s understanding of conservation (*n* = 384)

Determining the participant’s understanding of conservation is paramount, as this may impact their perceptions of conservation methods. When provided with four different meanings of conservation, 40% of the participants selected conservation as “protecting plants and animals.” In contrast, only 16% of participants believed that conservation means “isolating land for the exclusive use of plants and animals” (Fig. 5). The Vhembe community participants view conservation as a form of protection, not a tool to isolate land. Ideas of isolation and accessibility of protected areas are significant to the participants, as 85% of participants believe that nature reserves must be accessible to the local community. Additionally, 89% of participants believe that nature reserves can develop communities, improve infrastructure, attract tourists, generate employment opportunities, and alleviate poverty.

However, a disparity arises when considering the low local visitation numbers in the area. Only 51% of participants visit nature reserves (Table 3). More specifically, 45% of the population visited the Soutpansberg Mountain Range, 44% visited Mapungubwe National Park, and 31% visited the Kruger National Park (Table 3).

**Table 3:** Proportion of the population that visited nature reserves in the Vhembe District (*n* = 384)

The proportion of the population that visited:	Number	Percentage%
Local nature reserves in the area	196	51
The area around the Soutpansberg Mountain Range	174	45
The Mapungubwe National Park	171	44
The Kruger National Park	120	31

### Community perceptions of landscape initiatives

**Fig. 6:**
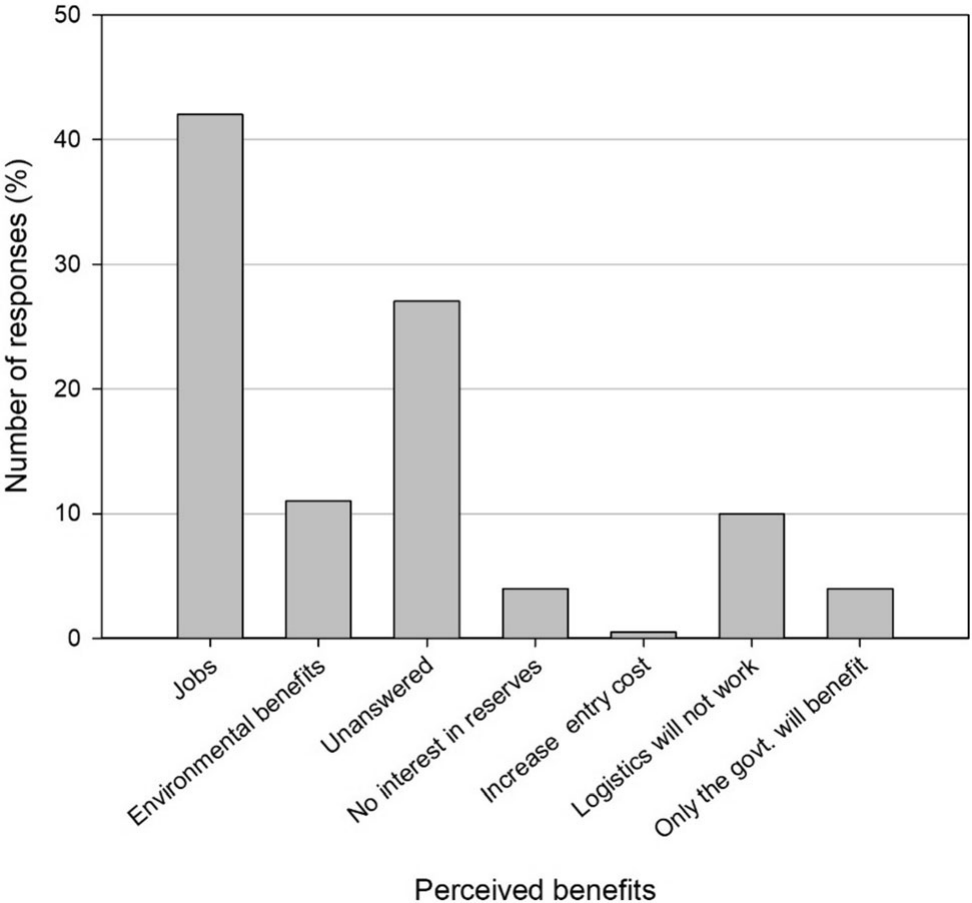
Community members’ perceived benefits of landscape initiatives in the Vhembe District (*n* = 384)

We explored local perceptions of corridors, revealing strong support for their implementation. A substantial 75% of participants expressed their support for linking nature reserves in the Vhembe District, with 77% expressing that this would yield direct benefits for them. Perceived benefits are highlighted in Fig. 6, with 42% of participants mentioning job creation, while only 11% cited environmental benefits. Interestingly, 77% of participants stated they would be open to working in the tourism or environmental sectors if jobs became available.

While most participants can envision perceived benefits from landscape initiatives, some locals harbor concerns. Some participants had no interest in reserves, were apprehensive about the possibility of an increase in entry costs, doubted the logistics of the corridor or network, and were skeptical about whether the locals or the government would benefit (Fig. 6). Notably, a high proportion of participants did not answer the question.

## Discussion

### Factors affecting landscape initiatives establishment

Several studies have presented criteria, checklists, models, and proposals for corridor and network expansion and creation (see, for example, Beier and Loe 1992; Fleury and Brown 1997; Gregory et al. 2021; Travers et al. 2021; Shafaghat et al. 2022; Chen et al. 2024). Our emphasis was directed toward understanding the social considerations of corridors.

The 15 interviewees were positive towards corridors, and the majority of the respondents (73%) were positive towards corridors, indicating a preference for landscape initiatives over traditional protected areas. However, several critical factors must be considered before implementing landscape initiatives. While the specifics may vary across landscapes, it is advisable for corridors and networks to encompass four or more protected areas, with corridors between reserves spanning over one kilometer. These physical parameters imply that landscape initiatives can cover extensive areas across landscapes, making contact with infrastructure inevitable. The Vhembe District is densely populated, with the latest figures estimating a population of 1.4 million (Limpopo Provincial Government 2023). While large sections of the region are natural, human settlements are prevalent along the district’s southern region and near Musina. The many roads in the area, as shown in Fig. 1, contribute to landscape fragmentation and impede future protected area connections. Roads may block wildlife movement and increase the number of vehicle-animal collisions (Garrah et al. 2015). The presence of roads between protected areas in the district poses a significant challenge to expanding and linking protected areas. Underpasses may need to be built, but they are costly. Therefore, policymakers and planners must account for the impact of road mortality and consider linking protected areas where connectivity is uninterrupted by developments or roads (Shafaghat et al. 2022).

In addition to the challenges posed by developments in the Vhembe District, funding constraints present another hurdle to establishing corridors and networks (Fig. 2). Corridor creation involves resource-intensive activities such as possible land acquisition, placing or removing fences or boundaries, planning, managing, and monitoring possible poaching, and developing recreational facilities (Brammer 2022). Funders or landowners may not see a return on their investment, especially since the efficacy of corridors can only be determined 10–50 years after their establishment (Gregory and Beier 2014). Funding constraints are not unique to the Vhembe District and will likely pose challenges wherever conservation corridors are established. However, directing funds toward conservation initiatives may prove impractical in rural areas with high levels of unemployment and poverty. Rural areas and regions experiencing significant unemployment rates should exercise caution before undertaking conservation endeavors that may incur more significant costs than benefits.

The fundamental principles of property rights underlie terrestrial conservation practices. Such practices often require partial property acquisition, which can be complex given potential disputes over land ownership (Rissman 2013). The Vhembe District poses an interesting case study, as 46 of the 68 protected areas in Vhembe are privately owned (Dalziel and Evans 2024). Consequently, corridors and networks in Vhembe may involve private and national partnerships, an endeavor that failed to garner interest in any of the three landowners interviewed.

Complicating matters, South Africa lacks legislation or policies that encourage, support, or guide conservation corridors (Dalziel and Evans 2023). This dearth of regulatory framework means corridors are not yet a well-established tool in South Africa, and practitioners may face hurdles when implementing them. South Africa’s protected area policies, such as the National Biodiversity Strategy and Action Plan and the National Protected Area Expansion Strategy, must be updated to encourage and guide the establishment of corridors and networks and stress the importance of including communities prior to implementation. Many of South Africa’s protected area policies have surpassed their intended review periods and require substantial revitalization.

The absence of supportive, guiding legislation, coupled with possible funding constraints, infrastructure and connectivity limitations, and land rights and jurisdiction complexities, collectively exacerbate the challenges in establishing corridors or networks.

### Accessibility and land use

South Africa’s conservation and protected area history is rooted in Colonialism and Apartheid. Colonialism created the idea that nature could only be conserved through dispossessing and excluding people (Martínez-Alier 2001). The notion of conservation through dispossession, created during Colonialism, was adopted during Apartheid, which saw the forced removal and exclusion of citizens to establish protected areas (Brooks, 2005; Carruthers 2008; Gewald et al. 2018). The Natives Land Acts 27 of 1913 and Act 18 of 1936 (Union of South Africa, 1913, 1936) reduced African landownership to only 13% of the land in South Africa (Cock and Fig. 2000; Ramutsindela 2004). South Africa’s conservation history is characterized by "double exclusion", coined by Cock and Fig. (2000), where the majority of the citizens were unable to visit reserves and were marginalized from decision-making. National parks continued to serve as symbols of exclusion. Today, ensuring access to nature reserves is critical.

Throughout the study, the theme of accessibility emerged in two fundamental forms. Firstly, community members emphasized the importance of having access to nature reserves. Only a minority of the participants perceive conservation as synonymous with isolation. A total of 85% of the participants expressed a desire for nature reserves to remain accessible to locals. Secondly, the findings underscored the significance of considering access to land utilized by local communities. In regions like the Vhembe District, where 87% of the participants use the land, isolating land may alienate the community from the environment and conservation efforts, tarnishing views of conservation practices.

Around half the participants visit nature reserves in Vhembe (Table 3), signifying a notable interest in environmental engagement. Additionally, findings indicate that residents are interested in nature reserves. Factors that create the desire to travel are known as travel motivations. Residents may visit nature reserves due to cultural significance, relaxation, recreation, experiencing wildlife, and socializing (Ma et al. 2018; Scholtz et al. 2013). Moreover, participants believe that nature reserves possess the potential to develop communities, upgrade infrastructure, attract tourists, alleviate poverty, and create jobs.

However, accessibility is limited as it seems they are not the target market for some of the existing nature reserves in Vhembe, especially the private reserves. Visitation numbers could surge if newly connected and established reserves specifically target the local demographic. Our findings show that only 31% of participants have visited the Kruger National Park, and only 44% visited the Mapungubwe National Park (Table 3). Considering the entry fees of nature reserves is essential, given that most of the population is in the Upper-Bound Poverty Line. Presently, the entry fees for these reserves stand at $6 and $3, respectively. When coupled with transportation expenses, visiting these reserves becomes prohibitive, considering that most people live on just over $2 a day. This financial burden renders visits to nature reserves a luxury for the community, excluding potential customers and residents. Corridors wield the potential to either involve or alienate communities from the land they once accessed, repeating the past. Without considering community perspectives, corridors may inadvertently reinforce the dominance of private landowners in the region. A paradigm shift is imperative in upcoming initiatives as locals are vital in facilitating effective long-term conservation (Mehta and Kellert 1998; Goldman 2009; Clerici et al. 2019; Neelakantan et al*.* 2021).

### Corridors and socio-economic development

Corridors and networks, especially those in South Africa, can potentially involve and benefit communities or exclude and isolate them. Social Impact Assessments (SIAs) are essential tools for understanding and mitigating the social consequences of development projects. The National Environmental Management Act (NEMA) 1998 (Act No. 107 of 1998) sets the framework for environmental management and assessments, requiring SIAs as part of the Environmental Impact Assessment (EIA) process for certain projects (Du Pisani and Sandham 2006). SIAs involve evaluating the potential impacts on local communities, including livelihood changes, to ensure that development is inclusive and sustainable (Hildebrandt and Sandham 2014). Corridors and networks should be considered a development and must be subject to a Social Impact Assessment before establishing them. This is essential to ensure the community will benefit.

Previous research suggests that conservation corridors have the potential to create employment opportunities (see Landry and Chirwa 2011; Bleyer et al. 2016). Corridors may attract tourists, creating employment in the retail and hospitality sectors. Moreover, corridors also require monitoring and management, which may create jobs, provide educational experiences, and improve local resources. Additionally, nature reserves that promote trophy hunting or focus on anti-poaching surveillance often employ local community members (Zafa-Calvo and Moreno-Penaranda, 2018). Infrastructure development, including fences and information centers, will create short-term employment opportunities.

In addition to job creation, conservation corridors might improve development in the area. Where roads do not interfere with linking protected areas, improved roads may facilitate connectivity and transportation (Ryan and Hartter 2012). In turn, this could stimulate economic activity and facilitate access to essential services and nature reserves. Corridors may spark increased education and environmental interest among locals as they serve as valuable educational resources. Training centers may be established requiring trainers, operators, and tour guides. Encouraging engagement between locals and the environment can further cultivate support for future projects—however, realizing these potential benefits hinges on whether locals can access these sites.

It remains challenging to precisely quantify the economic contribution and job creation facilitated by conservation corridors. Despite evidence suggesting potential social benefits, measuring the full extent of their impact remains elusive. Around a quarter of participants reported being unemployed, underscoring the significance of potential job creation associated with corridor development. Furthermore, our study revealed that respondents are open to work in the tourism or environmental sector. Given the prevailing conditions of extreme unemployment in the region, the prospect of job creation is appealing to locals.

However, the employment opportunities generated by corridors tend to be seasonal and limited, often engaging only a small portion of the population. Creating many jobs akin to those seen in major parks like the Kruger National Park requires large-scale corridors. Hinging on tourism for job creation is idealistic. Consequently, expecting a substantial surge in tourism numbers solely due to corridor establishment may be unrealistic. Given the persistently high unemployment rates in the region, the impact of corridors on employment in Vhembe may be limited. Areas with lower unemployment rates may garner more support for corridors due to their higher potential to increase employment rates. It is important to note that there are other costs of corridors not explicitly discussed in the paper. For example, conservation corridors and ecological networks may facilitate bushfires, accelerate the spread of diseases, expose wildlife to predators, and heighten the risk of poaching (Simberloff and Cox 1987).

### Limitations

This study employed interviews and surveys to collect data. Both data collection methods have various limitations. Sampling bias may occur if the survey does not accurately represent the target population. To mitigate this, efforts were made to calculate a representative sample (see Research method). Additionally, response bias must be considered, as participants may provide misleading information. The conditions under which the interview is conducted may also impact respondents’ answers (Fagarasanu and Kumar 2002). The questions asked may reflect the interviewer’s bias. Moreover, while surveys allow for a large volume of data to be collected, they often use closed-ended questions, which fail to capture the complexity of matters (Fowler 2013; Creswell and Creswell 2017). To mitigate potential overgeneralizations, broad statements were avoided. Although the representative sample accurately reflected the perspectives of the larger population, statements referring to “the community” pertain exclusively to those individuals who participated in this study.

## Conclusion

On paper, the Vhembe District is an ideal site for corridor and network establishment due to its numerous nature reserves, relatively unspoiled landscapes, and high natural value. The community members surveyed in the study also support conservation efforts. In addition, the Soutpansberg is earmarked as essential for conservation, with a preference for corridor-based approaches.

The community participants romanticize the idea of conservation corridors due to their potential to create jobs and aid regional development. However, the employment opportunities created might not align with the locals’ expectations. Some jobs created will be seasonal or limited to once-off construction jobs, offering only marginal relief to the current unemployment figures.

Additionally, findings suggest that establishing corridors necessitates careful consideration of other essential factors. Extensive road networks and densely populated towns pose obstacles to corridor creation. Infrastructure is not unique to Vhembe but is applicable wherever practitioners seek to establish corridors. Further, funding presents a significant challenge, as their creation demands investment in fencing, ecological planning, and monitoring. These expenses may not be easily justified, particularly when more pressing social issues take precedence. Moreover, the absence of clear legislative guidance in South Africa complicates matters further. Furthermore, participants emphasized the importance of accessibility. Any network created must undergo a Social Impact Assessment and consider the community as its primary target market. Failure to do so may result in community alienation, potentially damaging future support for environmental-related issues.

Landscape initiatives hold immense potential in the region. Corridors could protect vulnerable Key Biodiversity Areas, boost tourism, spur development, and offer employment opportunities. Yet, corridor proponents lack the necessary legislative support and guidance. Evaluating the above factors before establishing corridors or networks is imperative. As a result, we advocate for comprehensive corridor legislation and updated protected area policies to guarantee successful long-term conservation. These policies must require public participation in landscape initiative planning. Community involvement is particularly vital in rural areas, where residents anticipate that new opportunities may bring jobs to the area.

This study’s significance exceeds its regional context, offering insights of global relevance, especially in rural areas. Community engagement emerges as a critical factor regardless of where corridors are established. While challenges may vary from site to site, robust community engagement facilitates the proactive identification of possible issues. This study offers actionable insights that could be transferred to corridor endeavors in southern Africa and globally and underscores the necessity of planning that prioritizes community engagement.
